# A vehicle re-identification framework based on the improved multi-branch feature fusion network

**DOI:** 10.1038/s41598-021-99646-6

**Published:** 2021-10-12

**Authors:** Leilei Rong, Yan Xu, Xiaolei Zhou, Lisu Han, Linghui Li, Xuguang Pan

**Affiliations:** College of Electronic and Information Engineering, Shandong University of Science & Technology, Qingdao, 266590 China

**Keywords:** Electrical and electronic engineering, Computer science

## Abstract

Vehicle re-identification (re-id) aims to solve the problems of matching and identifying the same vehicle under the scenes across multiple surveillance cameras. For public security and intelligent transportation system (ITS), it is extremely important to locate the target vehicle quickly and accurately in the massive vehicle database. However, re-id of the target vehicle is very challenging due to many factors, such as the orientation variations, illumination changes, occlusion, low resolution, rapid vehicle movement, and amounts of similar vehicle models. In order to resolve the difficulties and enhance the accuracy for vehicle re-id, in this work, we propose an improved multi-branch network in which global–local feature fusion, channel attention mechanism and weighted local feature are comprehensively combined. Firstly, the fusion of global and local features is adopted to obtain more information of the vehicle and enhance the learning ability of the model; Secondly, the channel attention module in the feature extraction branch is embedded to extract the personalized features of the targeting vehicle; Finally, the background and noise information on feature extraction is controlled by weighted local feature. The results of comprehensive experiments on the mainstream evaluation datasets including VeRi-776, VRIC, and VehicleID indicate that our method can effectively improve the accuracy of vehicle re-identification and is superior to the state-of-the-art methods.

## Introduction

**Figure 1:**
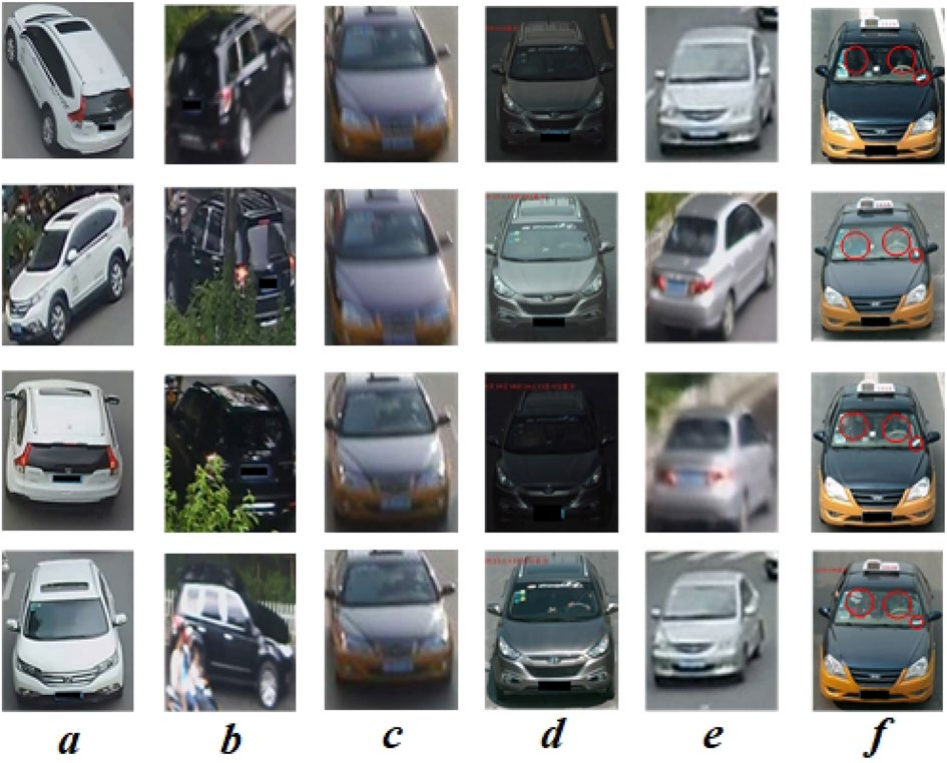
Illustration of challenges in vehicle re-id. The vehicle images(**a**–**e**) in each column are collected with the same vehicle, but their appearances are quite different due to various challenging factors, e.g., viewpoints, illumination, occlusion, low resolution and motion blur. The last column(**f**) illustrates the challenges of different vehicle identities with extremely similar appearance, where the red circles indicate the differences in local features.

Vehicle re-identification, an intelligent surveillance camera analysis technology, is indispensable to building smart and safe cities. Vehicle re-id is similar to pedestrian re-identification^1–5^, both of which belong to object re-identification, and are closely related to object recognition and fine-grained classification. The task of vehicle re-id is to retrieve a given vehicle among all gallery vehicle images captured across multiple surveillance cameras. However, it is challenging to do so due to various viewpoints, occlusion, motion blur, illumination, and low resolution, as shown in Fig. 1a–e. Furthermore, vehicle re-id is particularly difficult in that different vehicles may have similar or even the same appearance especially for those with the same model, as shown in Fig. 1f.

### Vehicle datasets

Liu et al.^6^ released the first vehicle dataset **VeRi-776** which contains 37,778 images of 576 vehicles as training set, 11,579 images of 200 vehicles as gallery set and 1678 images of 200 vehicles as query set. In addition to vehicle images, it also provides vehicles’ attributes (color and type) information and a part of license plate information. Liu et al.^7^ proposed a larger dataset **VehicleID** with 221,763 images of 26,267 vehicles from multiple real-world surveillance cameras, including the training set with 110,178 images of 13,134 vehicles and testing set with 111,585 images of 13,133 vehicles. More recently, Kanaci et al.^8^ introduced **VRIC**, a more realistic and challenging vehicle re-id benchmark which includes 54,808 images of 2811 vehicles as training set, 2811 images of 2811 vehicles as probe set and 2811 images of 2811 vehicles as gallery set.

### Vehicle re-id methods

In the field of re-id, the mainstream method is feature learning, whose principal task is to learn and extract more discriminative and robust vehicle features. For example, Zhu et al.^9^ proposed a Shortly and Densely convolutional neural Network (VRSDNet), which utilized a list of short and dense units (SDUs), necessary pooling, and spatial normalization layers to enhance the feature learning ability. Liu et al.^10^ encouraged the deep model to place emphasis on more details in local regions, so as to obtain more discriminative features. Cheng et al.^11^ introduced Multi-Scale Deep Feature Fusion Network (MSDeep) to conduct both multi-scale and multi-level features for precise vehicle re-id. Chen et al.^12^ extracted more robust and discriminative features via the view-aware feature learning aligning and enhancing common visible regions. Khorramshahi et al.^13^ presented a dual-path adaptive attention vehicle re-identification (AAVER) model, which is a robust end-to-end framework, combining macroscopic global features with localized discriminative features to efficiently identify a probe image in a gallery of varying sizes. Zheng et al.^14^ proposed a multi-scale attention framework (MSA) to fuse the discriminative local cues and effective global information. Wang et al.^15^ designed an attribute-guided network (AGNet) with attention module which could learn global representation with abundant attribute features in an end-to-end manner. He et al.^16^ used a simple and efficient part-regularized discriminative feature preserving method to improve the recognition ability of subtle information. Huang et al.^17^ introduced a Position-Dependent Deep Metric unit, which is capable of learning a similarity metric adaptive to local feature structure. Cui et al.^18^ designed a network that combined attention mechanisms and long short-term memory network (LSTM) for the recognition of spatial relations.

### Local feature

In the past, most vehicle re-id methods just used global features. Some detailed information are often ignored due to the limited scale and weak diversity of vehicle datasets. To solve this problem, the accuracy of re-identification has been improved by locating significant vehicle parts from images in many previous works^5,19,20^. Zhang et al.^21^ proposed a novel Part-Guided Attention Network (PGAN) for vehicle instance retrieval (IR) to extract part regions of each vehicle image from an object detection model. Khorramshahi et al.^22^ and Liu et al.^23^ highlighted the importance of attending to discriminative vehicle regions. Liu et al.^10^ explored a Region-Aware deep Model (RAM) to extract regional features from three overlapped local regions and pay more attention to the details in local regions. Suprem et al.^24^ presented global and local attention modules for re-identification (GLAMOR), which extracts additional global features and performs self-guided local feature extraction using global and local attention, respectively.

### Attention mechanism

Attention mechanism^25,26^ is widely implemented in various fields of deep learning and it has been employed in literature^27^ in vehicle re-identification field. Teng et al.^27^ proposed a spatial and channel attention network to mine the discriminative features in vehicle re-id task. As a kind of soft attention, channel attention mechanism’s final function is to give higher weight to areas containing different information. To this end, we introduce channel attention mechanism that can aggregate semantic similarity channels and attain more discriminative feature representations for vehicle re-id.

To extract more discriminative and robust features for vehicle images, we propose a vehicle re-id method based on global–local feature fusion, channel attention mechanism, and weighted local feature. We first choose ResNet-50 as the backbone network and construct three feature learning branches (Global Branch, Local Branch1, and Local Branch2) after res_conv5 layer. By fusing global and local features to obtain more complete information of the vehicle, the learning ability of the model is enhanced. In the second place, we insert the channel attention module in the Local Branch1 and the Local Branch2 so that the network can extract the personalized features of the vehicle. In the last place, the influence of background and noise information on feature extraction is weakened by weighted local feature. Finally, extensive experimental results on three vehicle datasets verify the promising performance of the proposed method compared to state-of-the-art methods.

## Our algorithm

**Figure 2:**
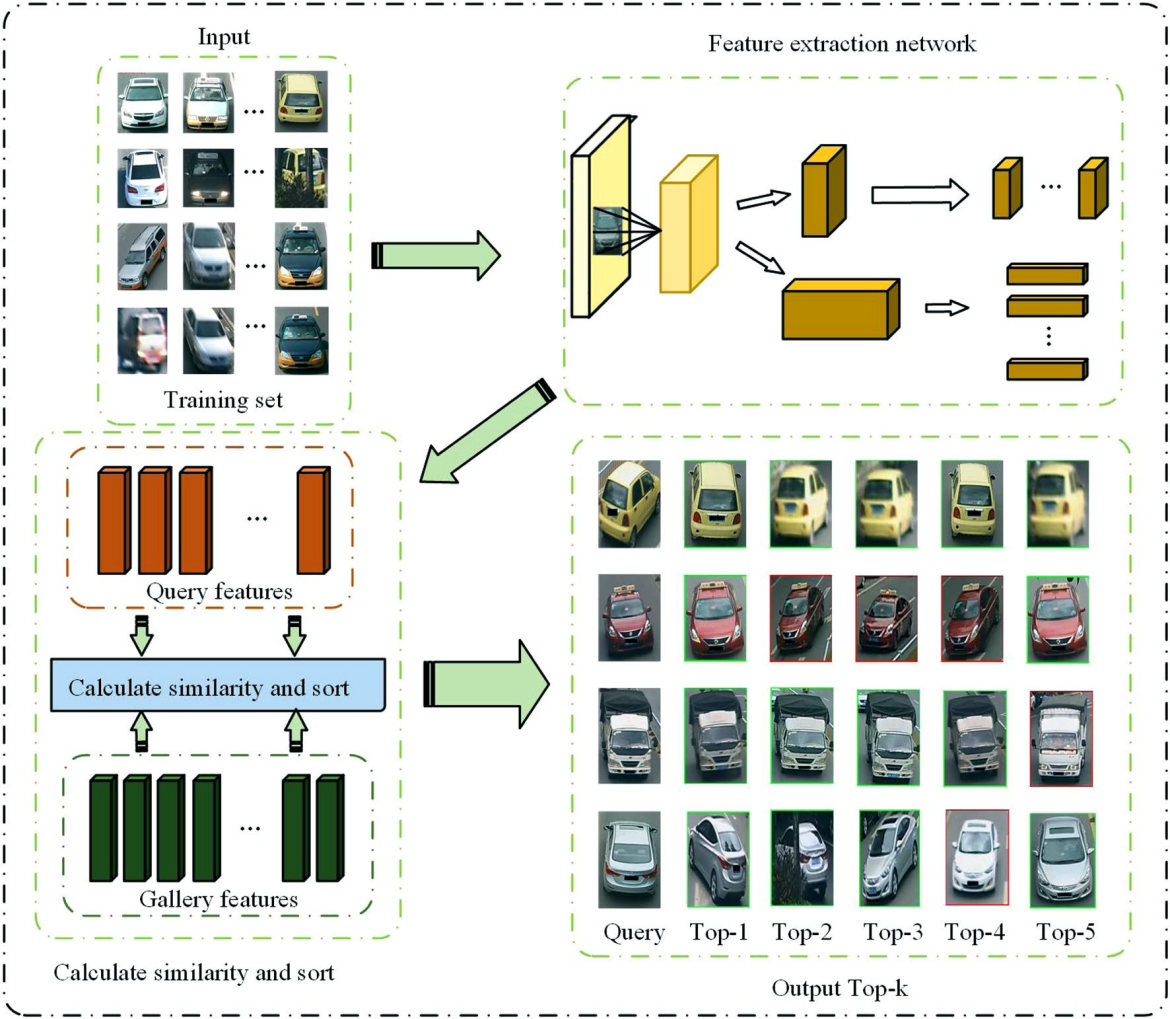
The overall framework of the algorithm model.

The algorithm model framework of this paper is shown in Fig. 2. Firstly, the proposed multi-branch network is used to extract vehicle features of training set. Then the similarity between Query and Gallery vehicle features is calculated. Finally, the similarity scores are sorted to obtain the retrieval results of all the vehicle images of Query in the Gallery.

### Multi-branch network architecture

**Figure 3:**
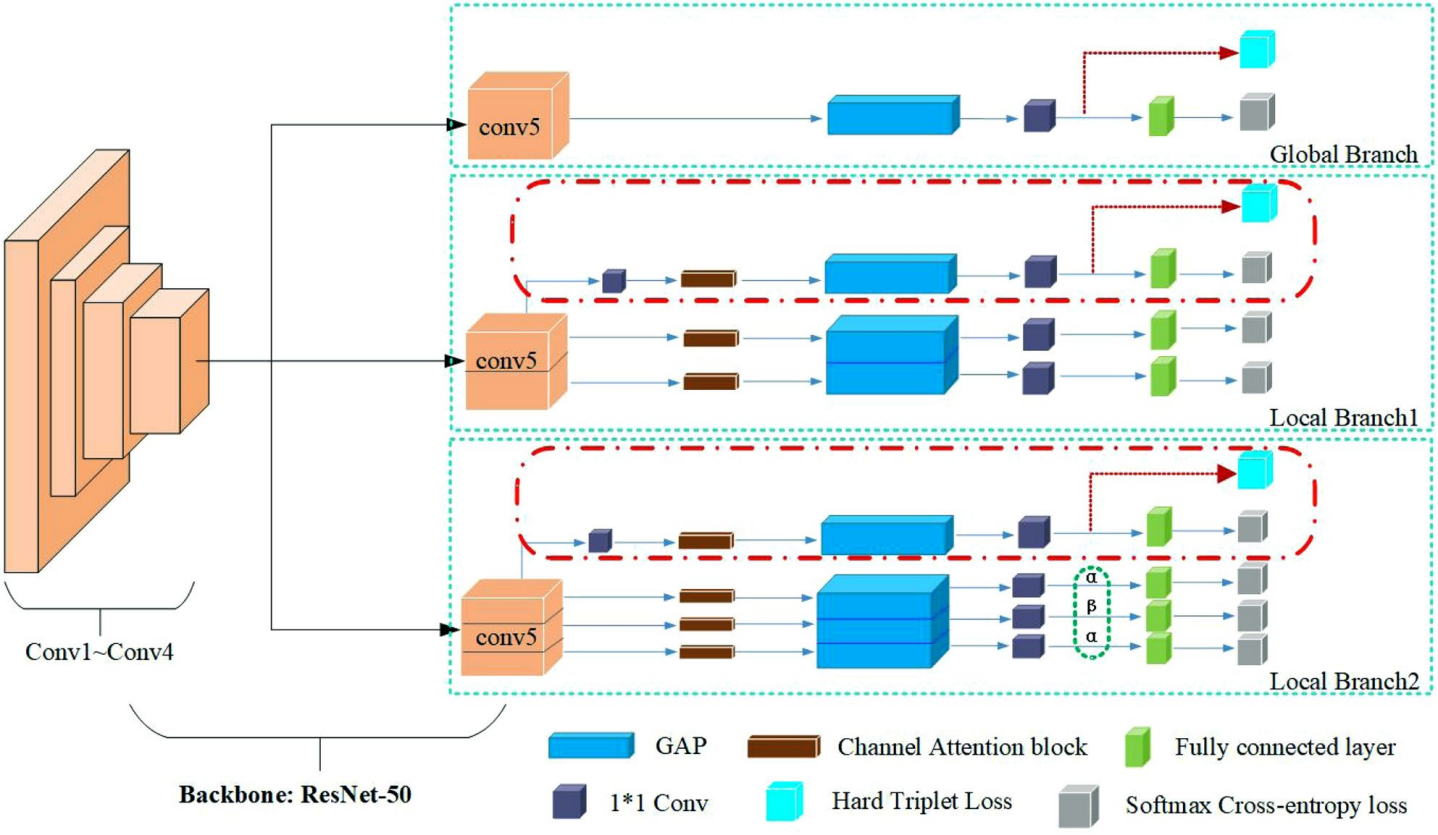
Multi-branch network architecture. GAP and 1*1 Conv refer to Global Average Pooling and 1*1 convolutional layer, respectively.

The architecture of multi-branch network is shown in Fig. 3. The first is a Global Branch, which learns the global feature representations without any partition information. The second and third are Local Branch1 and Local Branch2 respectively. They share a similar network architecture, and their difference is that the Local Branch1 divides the height of the feature map into two pieces, while the Local branch2 divides the height of the feature map into three parts. In particular, Local Branch1 and Local Branch2 all contain a global branch which aims to solve the problem of low robustness of learning local features by focusing on specific semantic regions.

In Local Branch1 and Local Branch2, we use the channel attention mechanism to give higher weight for important feature information. Global average pooling (GAP)^28^ is used to average each feature map and output a value. GAP replaces the fully connected layer and greatly reduces the number of parameters. It is worth mentioning that we also used a 1*1 convolution before the GAP block of the global branches of Local Branch1 and Local Branch2. This can not only reduce the number of channels, but also simplify calculations later. After the GAP block, 1*1 convolution block is used to increase the dimension, which can extract high dimensional features, and enhance the effect of feature extraction.

During the training, each branch trains separately and does not share the weight. But when testing, all branch information will be assembled into a comprehensive feature to improve network performance.

### Feature map segmentation

Research has shown that the discriminative features of vehicle are mainly concentrated in some local regions of the image^10,19–24^. In order to weaken the interference of noise and background and enhance the learning ability of the network, inspired by literature^19,20^, we adopt the approach of horizontal segmentation feature map.

As shown in Fig. 3, in Local Branch1 and Local Branch2, we adopt the idea of horizontal segmentation from coarse to fine, and divide the feature map into two and three parts respectively. Deep learning strategies can capture the best response area from the entire image. Therefore, feature extraction is performed on each image after segmentation, which can capture more fine-grained vehicle features.

### Weighted local feature

**Figure 4:**
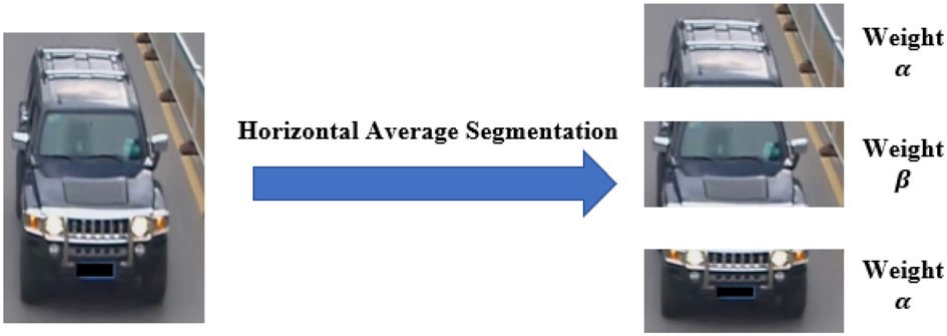
Segmentation and weighted vehicle image.

The vehicle usually locates in the middle of the image, the upper and lower parts of the image usually contain a lot of background information. Therefore, we assign the weight $\boldsymbol{\alpha }$ to the upper and lower parts of the image, and the weight of the middle part to ${\varvec{\beta}}$ ($\boldsymbol{\alpha }<{\varvec{\beta}}$), as shown in Fig. 4.

### Channel attention mechanism

**Figure 5:**
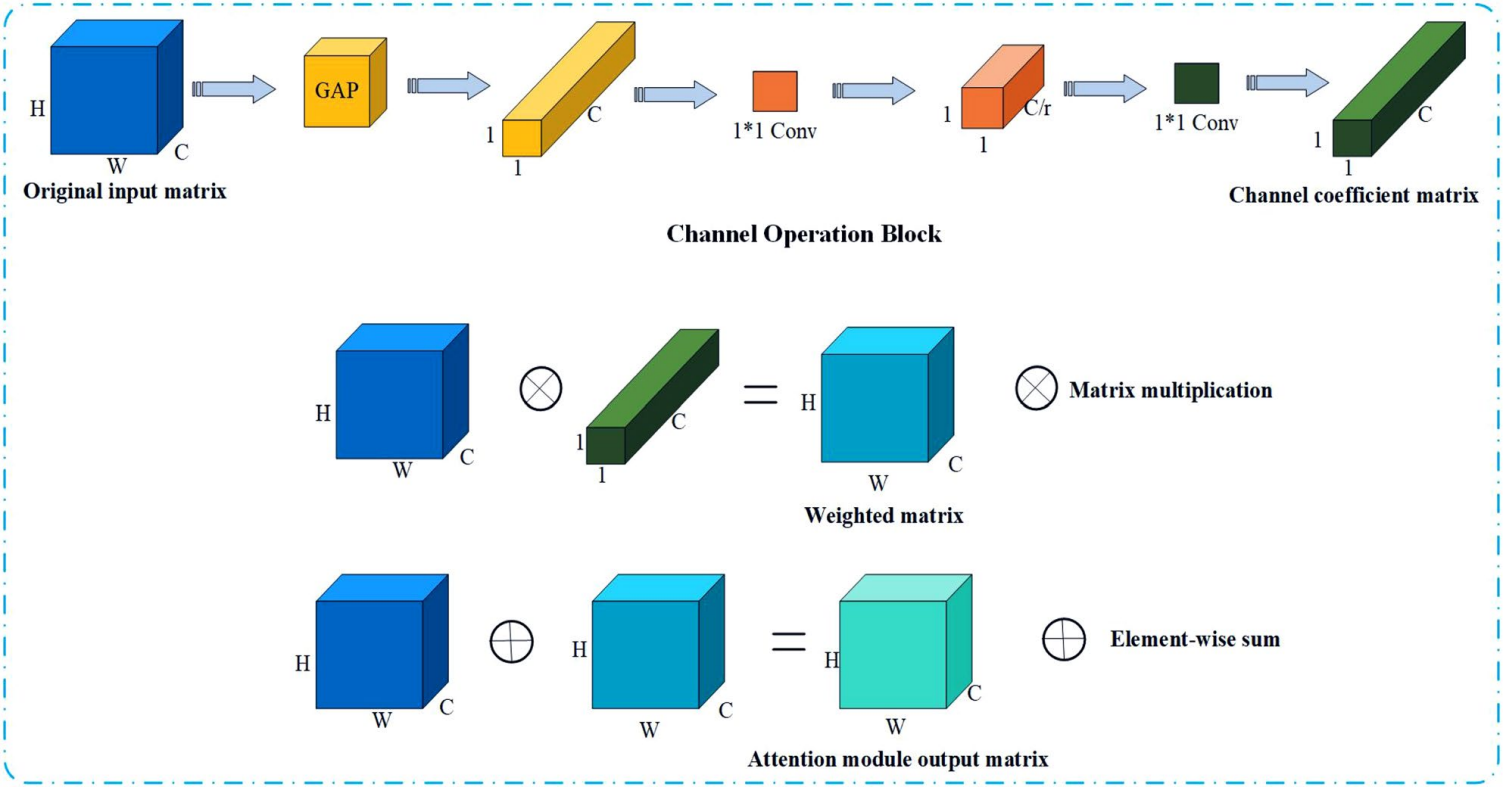
Channel Attention Module (CAM)^29^. $H$,$W$,$C$ represent the height, width, and channel number of the feature map respectively. $r$ is the scaling factor.

In addition to weighted local feature, we also introduce an attention module. This module can efficiently promote the network to extract the detailed features of the vehicle, such as windshield stickers, vehicle scratches. Figure 5 shows the channel attention module. The channel attention mechanism can be divided into three stages: channel operation stage, channel weighting stage, and channel superposition stage.

During the channel operation stage, the global average pooling is carried out on the original input matrix, so that the original input matrix with the dimension of H*W*C is changed into a channel descriptor of 1*1*C, which can reduce the computational cost and accelerate the network training speed. Then two 1*1 convolution modules are used to first reduce the dimension of channel descriptor and then increase the dimension. There is a dimensionality reduction factor $r$ between the two 1*1 convolution modules, and the dimension change is controlled by $r$. Finally, through the rise and fall of dimensions, the characteristic information of different channels is fused and the correlation between channels is captured to obtain a 1*1*C channel weight matrix. Then the original input matrix is multiplied by the channel weight matrix to get the weighted matrix, this process is called channel weighting stage. Finally, the output matrix of the attention module is obtained by adding the weighted matrix to the original input matrix in the channel superposition stage.

### Loss functions

In this paper, we introduce two loss functions: Softmax cross-entropy loss^19^ and hard mining triplet loss^30^. The total loss combining Softmax cross-entropy loss with hard mining triplet loss is used to our training experiment. The loss can be described as:

1 $$L_{Softmax} = - \mathop \sum \limits_{i = 1}^{{N_{i} }} \log \left( {\frac{{\exp \left( {x_{y} } \right)}}{{\mathop \sum \nolimits_{j = 1}^{{N_{id} }} \exp (x_{j} )}}} \right)$$

2 $$L_{hard\,mining\,triplet} = \sum\limits_{i = 1}^{Q} {\sum\limits_{A = 1}^{K} {\left[ {\overbrace {{\mathop {max}\limits_{P = 1, \ldots ,K} \left\| {A_{i} - P_{i} } \right\|_{2} }}^{hardest\,positive} - \overbrace {{\mathop {max}\limits_{\begin{subarray}{l} N = 1, \ldots ,K \\ j = 1, \ldots ,Q \\ i \ne j \end{subarray} } \left\| {A_{i} - N_{j} } \right\|_{2} + \delta }}^{hardest\,negative}} \right]_{ + } } }$$

3 $$L_{total} = L_{Softmax} + \lambda *L_{hard\,mining\,triplet}$$

where the meanings of the variates of (1), (2) and (3) are listed in Table 1.

**Table 1:** The variate and meaning of loss function.

Variate	Meaning
${N}_{i}$	The number of vehicle images per batch
${N}_{id}$	The number of vehicle identities
${x}_{j}$	The output of fully connected layer for $j$ th identity
$y$	The ground truth identity of input vehicle image
${A}_{i}$	Anchor
${P}_{i}$	Positive
${N}_{i}$	Negative
$\delta$	Minimal margin
$\lambda$	Weight

## Experiment results and discussion

To evaluate the performance of our model, we conduct experiments on three large-scale vehicle re-id datasets: VeRi-776, VRIC, and VehicleID. Firstly, we report a set of ablation studies (mainly on VeRi-776) to validate the effectiveness of each component. Secondly, we compare the performance of our model against existing state-of-the-art methods on three datasets. Finally, we discuss how our model achieves its effectiveness.

### Implementation details and evaluation metric

In our experiments, the software tools are *PyTorch*, *CUDA11.1*, and *CUDNN V8.0.4.30*. The hardware device is a workstation equipped with *AMD Ryzen 5 3600X CPU 32G*, *NVIDIA GeForce RTX 3080* and 256 GB + 2 TB memory. During training, the input images are re-sized to 384*128 and then augmented by random horizontal flip, normalization, and random erasing. We set the training batch size to 32, the initial learning rate is 3*${10}^{-4}$, and the learning rate decreases to 0.1 times at 20th and 40th epoch. At the same time, we choose the *AMSGrad* optimizer to train the network. The testing images are re-sized to 384*128 and augmented only by normalization. The weight of Local Branch 2 is 0.3 for $\boldsymbol{\alpha }$ and 0.4 for ${\varvec{\beta}}$. After many experiments, the attenuation factor ${\varvec{r}}$ of the channel attention module is set to 4. The margin $\delta$ in triplet loss is set to 1.2 in all experiments and the parameter $\lambda$ in total loss is set to 0.1.

Following the evaluation protocol of re-identification work^6,31,32^, we utilize the mean average precision (mAP) and Rank-$n$ (the expected correct matching pair in the top $n$ matches) as the evaluation metrics.

### Ablation experiments

#### Feature map segmentation setup

The feature map segmentation plays an extremely important role in local fine-grained feature extraction. By segmenting the feature map, the network can pay more attention to the fine-grained features of one local area and filter out the interference information in other areas. In terms of local feature extraction, we adopt a coarser to finer strategy, which is completed by Local branch 1 and Local branch 2 respectively. To verify the effectiveness of our segmentation feature map settings on the two local branches, we conduct ablation experiments on VeRi-776 dataset. As shown in Table 2, the effect of horizontal segmentation is much better than that of vertical segmentation. And in the horizontal segmentation setup, the best recognition effect is that the feature map is divided into two parts in Local branch 1 and three parts in Local branch 2.

**Table 2:** The results of different feature map segmentation setup.

Equally-split direction	Local branch 1	Local branch 2	mAP	Rank1
Vertical	2	3	43.51	78.10
	2	4	41.07	70.82
	3	4	39.11	67.99
Horizontal	2	3	**63.90**	**90.82**
	2	4	61.81	88.06
	3	4	57.98	85.31

Bold indicate the best results for the corresponding metrics.

#### Weight coefficient setup

Extensive analysis shows that, in most cases, the discriminative features of vehicles are mainly located in the middle region of the image, and the upper and lower of the image contain little vehicle information. Therefore, in Local Branch 2, the feature map is divided horizontally into three parts. Meanwhile, the upper and lower parts are given a small weight $\boldsymbol{\alpha }$, while the middle part is given a large weight ${\varvec{\beta}}$. For the specific values of weights $\boldsymbol{\alpha }$ and ${\varvec{\beta}}$, we conduct experiments on VeRi-776 dataset. As can be seen from Table 3, when the setting of $\boldsymbol{\alpha }$ and ${\varvec{\beta}}$ is 0.3 and 0.4, the detection results are the best.

**Table 3:** The results of different weight coefficient setup.

$\boldsymbol{\alpha }$	${\varvec{\beta}}$	mAP	Rank-1
0.2	0.4	74.01	95.33
0.2	0.5	74.89	95.22
0.2	0.6	75.14	95.28
**0.3**	**0.4**	**77.12**	**96.30**
0.3	0.5	72.19	94.50

Bold indicate the best results for the corresponding metrics.

#### Multi-branch network architecture

We choose ResNet-50 with the global feature branch as the baseline. Seven variants are then constructed based on the baseline (***Best view in color.***):

- Baseline + Local Branch1(no red dotted area) + CAM;
- Baseline + Local Branch2(no red dotted area) + CAM;
- Local Branch1(no red dotted area) + Local Branch2(no red dotted area and green dotted area);
- Local Branch1(no red dotted area) + Local Branch2(no red dotted area) + Green dotted area;
- Local Branch1(no red dotted area) + Local Branch2(no red dotted area) + Green dotted area + CAM;
- Baseline + Local Branch1(no red dotted area) + Local Branch2(no red dotted area) + Green dotted area + CAM;
- Baseline + Local Branch1 + Local Branch2 + Green dotted area + CAM.

The detailed results of the ablation studies on VeRi-776 dataset are illustrated in Table 4.

**Table 4:** The ablation studies on VeRi-776 dataset (in %).

Method	VeRi-776	
	mAP	Rank-1
Baseline	69.18	93.21
(a) Baseline + Local Branch1(no red dotted area) + CAM	72.13	94.04
(b) Baseline + Local Branch2(no red dotted area) + CAM	73.05	94.16
(c) Local Branch1(no red dotted area) + Local Branch2(no red dotted area and green dotted area)	63.90	90.82
(d) Local Branch1(no red dotted area) + Local Branch2(no red dotted area) + Green dotted area	69.57	91.66
(e) Local Branch1(no red dotted area) + Local Branch2(no red dotted area) + Green dotted area + CAM	71.17	93.86
(f) Baseline + Local Branch1(no red dotted area) + Local Branch2(no red dotted area) + Green dotted area + CAM	76.32	95.83
(g) Baseline + Local Branch1 + Local Branch2 + Green dotted area + CAM (**Ours**)	**77.12**	**96.30**

Bold indicate the best results for the corresponding metrics.

**Figure 6:**
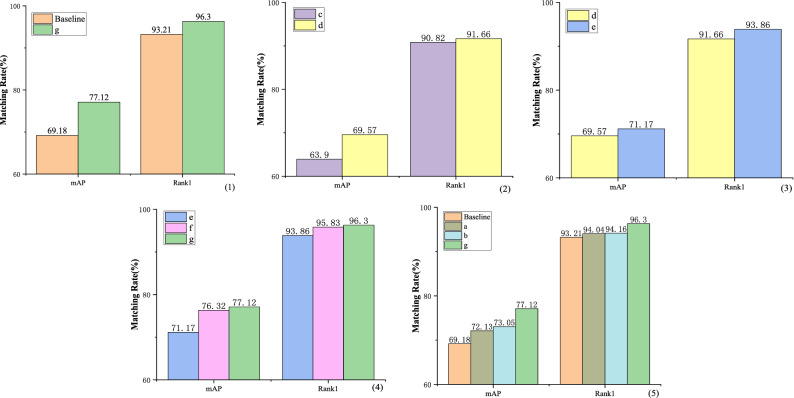
Ablation Experiment of the proposed framework on VeRi-776 dataset (in %).

It can be observed from Table 4 and Fig. 6(1) that compared with the baseline network, our improved network has increased by 7.94% and 3.09% on mAP and Rank-1 respectively. It proves that our network has strong robustness.

Compared with network ***c***, network ***d*** performs weighting processing on local features, and mAP is improved by 5.67%, which proves the effectiveness of weighting processing, as shown in Fig. 6(2). In Fig. 6(3), compared with network ***d***, mAP and Rank-1 of network ***e*** are improved by 1.60% and 2.20% respectively after adding channel attention block. Figure 6(4) shows that by compared with the experimental results of networks (***e***, ***f* and *g*)**, the importance of global features can be proved.

As shown in Fig. 6(5), comparing the baseline network, network ***a*** and network ***b*** with our improved network, we can draw two conclusions: first, combining global and local features can greatly improve the recognition accuracy; second, better recognition effect can be achieved by using feature map segmentation to fully extract vehicle local features from coarse to fine.

## Performance comparison with state-of-the-art methods

We compare our proposed method with multiple state-of-the-art vehicle re-identification approaches on three mainstream datasets, i.e., VeRi-776, VRIC, and VehicleID with corresponding evaluation metrics (mAP and Rank-n).

### Results on VeRi-776 dataset

Following the literature^31^ on standard evaluation, a test is conducted on the VeRi-776 dataset. Table 5 presents the results of comparisons between current state-of-the-art methods^9,10,13–15,33–40^ and our model on VeRi-776 dataset. Our proposed method achieves 96.30% on Rank-1 accuracy, 98.11% on Rank-5 accuracy and 77.12% on mAP without re-ranking. These results surpass current state-of-the-art models on almost all three metrics, especially on mAP. In this paper, our method only relies on the supervised information of ID, while VGG + C + T^33^, GS-TRE^34^, VAMI + ST^35^ and AGNet-ASL + STR^15^ exploit spatial–temporal information, and other methods also utilize extra annotations, but the accuracy of our model still exceeds all others. A good mAP score demonstrates that our model has a stronger potential to retrieve all the corresponding images of the same identity in the gallery set.

**Table 5:** The mAP, Rank-1 and Rank-5 on VeRi-776 dataset (in %).

Method	mAP	Rank-1	Rank-5	References
VRSDNet^9^	53.45	83.49	92.55	Multimed Tools Appl 2019
VGG + C + T^33^	58.78	86.41	92.91	ICME 2017
GS-TRE^34^	59.47	96.24	**98.97**	IEEE TMM 2018
AAVER^13^	61.18	88.97	94.70	ICCV 2019
VAMI + ST^35^	61.32	85.92	91.84	CVPR 2018
RAM^10^	61.50	88.60	94.00	ICME 2018
GRF + GGL^38^	61.7	89.4	95.0	CVPR 2018
QD-DLF^36^	61.83	88.50	94.46	IEEE TITS 2019
MSA^14^	62.89	92.07	96.19	Neural Computing and Applications 2020
SPAN w/ CPDM^40^	68.9	94.0	97.6	ECCV 2020
TCL + SL^37^	68.97	93.92	97.44	IEEE TIP 2019
AGNet-ASL + STR^15^	71.59	95.61	96.56	arXiv 2020
UMTS^39^	75.9	95.8	N/A	AAAI 2020
**Ours**	**77.12**	**96.30**	98.11	**Proposed**

Bold indicate the best results for the corresponding metrics.

N/A indicates that no data is provided.

### Results on VRIC dataset

VRIC is a relatively newly released dataset, hence, few results have been reported about it. For VRIC dataset, the test is conducted following the standard evaluation^8^. We compare the results of our proposed method with other models^8¸21,24,38^ on VRIC dataset. As shown in Table 6, by comparison, we can find out that our model outperforms the latest method^24^ by 1.39% in Rank-1 and 0.46% in Rank-5, respectively, and significantly improves the recognition effect of vehicle re-identification on both Rank-1 and Rank-5 accuracy.

**Table 6:** The mAP, Rank-1 and Rank-5 on VRIC dataset (in %).

Method	mAP	Rank-1	Rank-5	References
MSVF^8^	47.50	46.61	65.58	arXiv 2018
GRF + GGL^38^	71.66	63.68	81.85	CVPR 2018
PGAN^21^	**84.80**	78.00	93.20	arXiv 2020
GLAMOR^24^	76.48	78.58	93.63	arXiv 2020
**Ours**	82.75	**79.97**	**94.09**	**Proposed**

Bold indicate the best results for the corresponding metrics.

### Results on VehicleID dataset

For VehicleID dataset, all the tests are conducted following the standard evaluation^7^. Generally speaking, larger testing sets (1600 and 2400 test size) introduce more challenging and complex scenarios in real life, therefore, most methods perform better on smaller size (800) testing set. Table 7 shows our model outperforms other methods^9,10,13–15,33–40^ in all testing sets (800, 1600, and 2400 test size), and improves about 4.0% in mAP, Rank-1, and Rank-5 on all three testing sets, compared with the second-best methods achieved by AAVER^13^ and MSA^14^, respectively. These results demonstrate the robustness and superiority of our method.

**Table 7:** The mAP, Rank-1, and Rank-5 on VehiceID dataset (in %).

Method	Test800			Test1¸600			Test2¸400			References
	mAP	Rank1	Rank5	mAP	Rank1	Rank5	mAP	Rank1	Rank5	
VRSDNet^9^	63.52	56.98	86.90	57.07	50.57	80.05	49.68	42.92	73.44	Multimed Tools Appl 2019
VAMI^35^	N/A	63.12	83.25	N/A	52.87	75.12	N/A	47.34	70.29	CVPR 2018
VGG + C + T + S^33^	N/A	69.90	87.30	N/A	66.20	82.30	N/A	63.20	79.40	ICME 2017
AGNet-ASL^15^	74.05	71.15	83.78	72.08	69.23	81.41	69.66	65.74	78.28	arXiv 2020
GS-TRE^34^	75.40	75.90	84.20	74.30	74.80	83.60	72.40	74.00	82.70	IEEE TMM 2018
QD-DLF^36^	76.54	72.32	92.48	74.63	70.66	88.90	68.41	64.14	83.37	IEEE TITS 2019
AAVER^13^	N/A	74.69	93.82	N/A	68.62	89.95	N/A	63.54	85.64	ICCV 2019
RAM^10^	N/A	75.20	91.50	N/A	72.30	87.00	N/A	67.70	84.50	ICME 2018
TCL + SL^37^	80.13	74.97	87.44	77.26	72.84	81.98	75.25	71.20	79.29	CVPR 2018
GRF + GGL^38^	N/A	77.1	92.8	N/A	72.7	89.2	N/A	70.0	87.1	IEEE TIP 2019
MSA^14^	80.31	77.55	90.50	77.11	74.41	86.26	75.55	72.91	84.35	Neural Computing and Applications 2020
**Ours**	**87.70**	**81.96**	**95.35**	**84.26**	**77.85**	**92.44**	**80.87**	**74.07**	**89.55**	**Proposed**

Bold indicate the best results for the corresponding metrics.

N/A indicates that no data is provided.

### Discussion

In this paper, the approaches of global–local feature fusion, channel attention mechanism, and weighted local feature are introduced into our vehicle re-id framework to obtain more rapid and accurate results. The problem-solving pattern is close to those reported in related literature^5^. The main idea of this paper is to realize a robust feature learning network which takes the advantage of advanced methods to make full use of vehicle appearance attributes, and finally achieve good re-id effect; Previous literature^5,23,40^ mainly uses the method of target feature alignment to adjust the images to the same scale. This approach can reduce the intra-class differences and facilitate the comparison between target features, and finally simplify the subsequent re-id task. By contrast, our vehicle re-id model can not only accurately identify the same vehicle, but also effectively deal with various vehicle challenges in real life.

Beyond that, it can also be adopted to re-identify other rigid and large target objects under urban surveillance cameras, such as non-motorized vehicle re-identification, etc. This technology provides important technical support for intelligent transportation system and the construction of smart and safe cities.

#### Computation time

Our model has achieved good recognition results on three mainstream datasets. However, in real-world applications, accuracy is just one index for performance evaluation of a model. In re-id task, the computation time for the model is critical and nonnegligible for practical usage. Hence, we analyze the training epochs required by different models to converge to stable values. Taking VeRi-776 dataset as an example, the comparison results are shown in Table 8. Compared with those methods^22,23,40,41^, our model needs the least number of training epochs to achieve convergence, that is, our method is the most efficient in training stage. At the same time, we also calculate our training and inference time, as shown in Table 9.

**Table 8:** Comparison of training efficiency of different methods.

Method	Training Epochs	Rank-1
PVEN^40^	150	95.6
SAVER^22^	120	96.4
PCRNet^23^	100	95.4
VehicleNet^41^	72	**96.78**
**Ours**	**60**	96.30

Bold indicate the best results for the corresponding metrics.

**Table 9:** Training and inference computation times of our model for the three vehicle datasets.

Dataset	VeRi-776	VRIC	VehicleID
Training time	6.3 h	8.73 h	10.19 h
Inference time	0.4349 s	0.2240 s	0.8318 s

Inference time = TestingSize(img) ÷ BatchSize(img) × BatchTime(s).

### Visualization of model retrieval results

**Figure 7:**
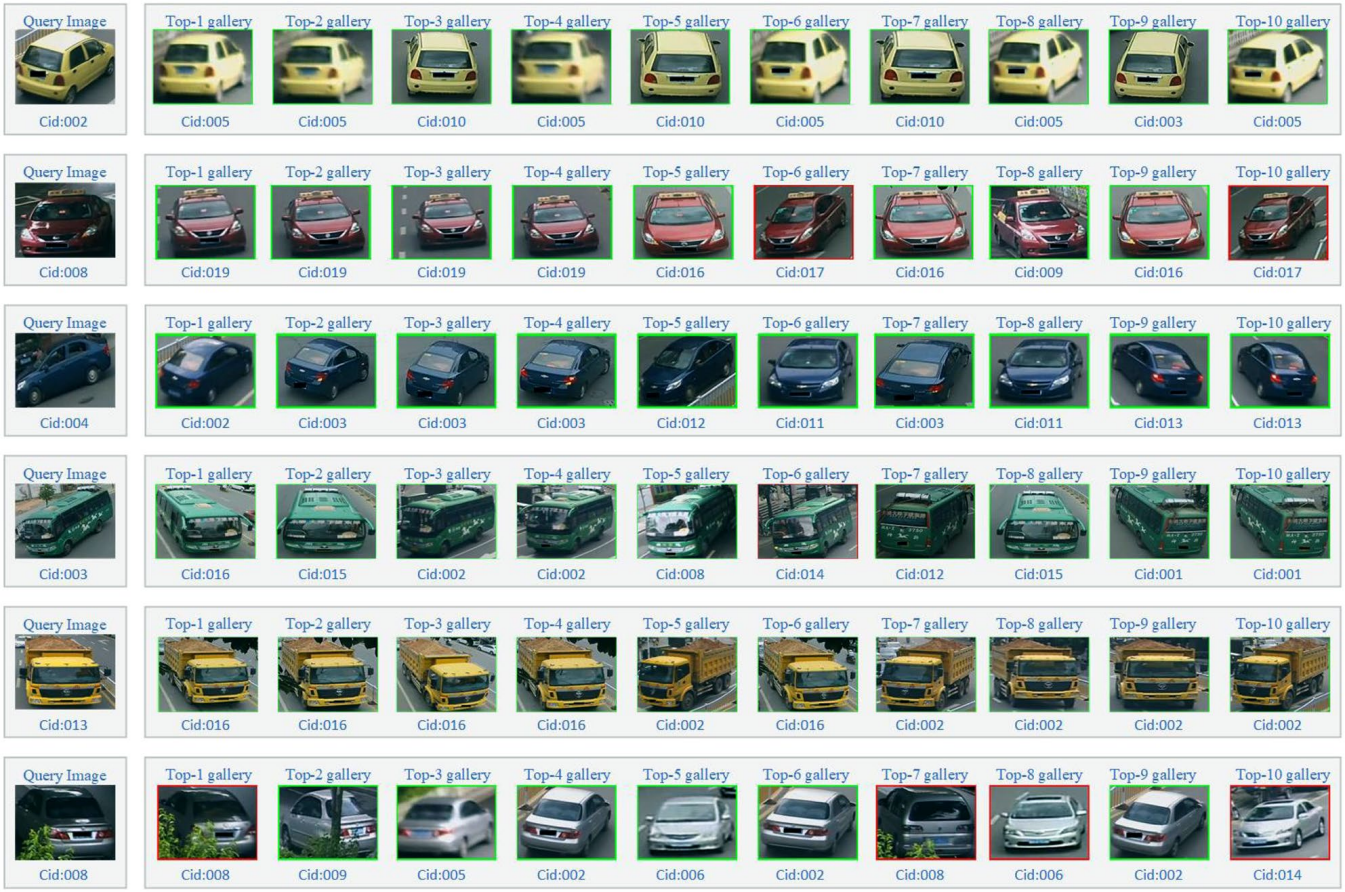
Visualization of model retrieval results.

To verify the retrieval ability of the model, we make visual processing on the retrieval results of the model, as shown in Fig. 7. The first column represents the target vehicle in Query set, and the other columns represent the retrieval results from Gallery set (the retrieval times are set to 10). Red border vehicle represents an incorrect retrieval and Green represents a correct retrieval. We can see that our model is robust to the challenges (e.g., viewpoints, occlusion, low resolution).

## Conclusion and future work

In this work, we propose a multi-branch network for vehicle re-identification. First of all, a channel attention mechanism strategy integrates discriminative information with global and local features. At the same time, feature extraction is optimized through attention mechanism and weighted local feature, so that more discriminative features are extracted. Results of extensive comparative evaluations have indicated that our method not only exceeds state-of-the-art results on three challenging vehicle re-id datasets, but also pushes the performance to an exceptional level.

At present, most of the deep learning algorithms are supervised learning, which requires a large number of annotations of datasets in the early stage. Unsupervised learning has been studied in many fields. Future vehicle re-identification field studies need to explore the related algorithms of unsupervised learning, which can greatly reduce the calibration of datasets and improve the utilization rate of vehicle images.
